# Development of a Framework for the Enrollment of Children and Families in Emergency Department Research

**DOI:** 10.1016/j.acepjo.2024.100018

**Published:** 2025-01-09

**Authors:** Aliya Redd, Rosemarie Fernandez, Diego Maldonado-Puebla, Julia Mortensen, Meredith Thompson, Antionette McFarlane, Colleen Gutman

**Affiliations:** 1 Department of Emergency Medicine, University of Florida College of Medicine, Gainesville, Gainesville, Florida, USA; 2 Center for Experiential Learning and Simulation, University of Florida College of Medicine, Gainesville, Florida, USA; 3 Department of Pediatrics, University of Florida College of Medicine, Gainesville, Florida, USA

**Keywords:** pediatric, research, emergency medicine

## Abstract

**Objectives:**

There remains a need to establish best practices for ethical and inclusive enrollment of children in emergency settings outside of established research networks. We aimed to develop a stakeholder-informed framework for enrolling children and families in emergency department (ED) research.

**Methods:**

We conducted a cross-sectional mixed-methods study using a convergent parallel design. Data collection tools were formulated by a multidisciplinary team. We approached caregivers of pediatric ED patients at a single site to complete a tablet-based questionnaire. We conducted 4-item structured interviews with (1) clinicians in a single pediatric ED and (2) pediatric ED researchers from across the United States. We calculated descriptive statistics for quantitative data and conducted a content analysis of qualitative data. These data were merged to develop a framework to support research recruitment and enrollment in the pediatric ED.

**Results:**

The questionnaire was completed by 225 caregivers (61% response). Caregivers’ likelihood of participating in research was high but varied by type of research, perceived importance, and the clinical context. Researchers (n = 11) and clinicians (n = 8) identified barriers to and facilitators of research recruitment in the ED at the caregiver, clinician, and systems level. Merging these data, we developed a framework of 5 principles: (1) high-quality communication is essential; (2) discussions about research participation cannot be coercive; (3) partnership with clinical teams is necessary; (4) research teams must adapt to unpredictability; and (5) participation in research may be viewed as an opportunity by families.

**Conclusion:**

Our framework provides key considerations for conducting ED research with children and families.

**The Bottom Line**

By using multiple methods to engage experts and key stakeholders, we developed a framework that highlights key considerations and areas in need of future study to guide researchers and institutions in conducting emergency department (ED) research with children and families.

## 1 Introduction

### 1.1 Background

Engaging children and families in research is essential for improving child health, yet there are unique ethical and pragmatic concerns when enrolling children in research.^1^^,^^2^ This is of particular importance for research conducted in the emergency setting.^3^^,^^4^ In addition to providing opportunities to improve care for acutely ill and injured children, pediatric research in the emergency department (ED) is necessary to promote health equity.^5^ As a safety net in the health care system, children who are cared for in the ED without adequate primary care access may otherwise have few opportunities to benefit from research participation.^6^ However, the process of research enrollment in the ED is distinct from that in an outpatient setting. ED visits are unplanned, patients do not have existing trust relationships with clinicians, the decision of whether to participate in research is time-sensitive, caregivers are often facing high levels of stress and uncertainty, and given the potential for high illness severity, clinical assessment and stabilization necessarily take precedence.^7^, ^8^, ^9^

### 1.2 Importance

Despite these challenges, caregivers are willing to participate in research, and several research networks have been successful in advancing the science of child health through clinical trials in the pediatric ED.^2^^,^^10^ Yet despite the growth of research in academic pediatric EDs, the majority of children seeking emergency care are evaluated in community EDs. This setting typically has a different model for clinical care delivery.^11^ Conducting pediatric ED research in such settings may enhance the generalizability and dissemination of research findings. However, efforts to do so may be hindered by a lack of research support infrastructure and low Institutional Review Board (IRB) familiarity with the unique needs of conducting research with children and families in emergency settings.^12^ As such, there continues to be a need to establish and disseminate best practices for enhancing ethical, just, and inclusive enrollment of children in pragmatic settings outside of established research networks.

### 1.3 Goals of This Investigation

We aimed to develop a pragmatic framework for enrolling children and families in ED research that reflects clinician, researcher, and family stakeholder input.

## 2 Methods

### 2.1 Overall Study Design, Participants, and Setting

In this mixed-methods research, we used a convergent parallel design to collect cross-sectional input from key stakeholders and experts to inform the development of a pragmatic framework for enrolling children and families in pediatric ED research.^13^ Key stakeholders included caregivers of patients and clinicians from the UF Health Shands Pediatric Emergency Department, an academic pediatric ED with approximately 26,000 annual patient visits. The UF pediatric ED is not currently affiliated with any research networks and had not previously conducted research requiring patient or caregiver enrollment prior to 2023. Experts included researchers and research coordinators with experience conducting pediatric ED research at academic medical centers across the United States. Key stakeholders and experts were recruited, and data were collected between June and August 2023. Our study was guided by the consensus-based checklist for reporting of survey studies. This study was approved by the University of Florida IRB.

### 2.2 Caregivers

We recruited a convenience sample of caregivers (18 years or older) of pediatric ED patients from the UF Health Shands Pediatric Emergency Department for a tablet-based questionnaire assessing preferences for research recruitment in the pediatric ED. Caregivers were not approached if (1) the patient was critically ill and undergoing active resuscitation, (2) the patient presented with an acute mental health concern, or (3) law enforcement officers were present. Eligible caregivers were approached when a research team member was present, which occurred at various times between 10:00 am and midnight over five 5-week periods in June and July 2023. Caregivers were approached at any time while in the ED to complete a brief online questionnaire using a research tablet or by scanning a quick response code for access on their personal devices. The questionnaire contained 29 closed-ended items and 1 open-ended item and took approximately 5 to 10 minutes to complete (Supplementary Appendix 1). Participants entered responses directly into a REDCap database hosted at the University of Florida.^14^ The questionnaire was available in English; caregivers who used languages other than English could complete the survey verbally with professional interpreting. The questionnaire was developed by a team with expertise in emergency medicine and pediatric research and equitable research enrollment (CG, RF, AM, and AR). Items were based on existing literature when possible and revised based on pilot testing with a sample of 13 caregivers recruited in the same manner as the study sample.^9^^,^^15^

### 2.3 Clinicians, Researchers, and Research Coordinators

We recruited a convenience sample of clinicians (attending and resident physicians, nurses, technicians, and support staff), researchers, and research coordinators who were aged 18 years or older. We approached clinicians in person at the UF Health Shands Pediatric Emergency Department for a 4-item structured interview assessing perceived barriers and facilitators of research enrollment in the pediatric ED (Supplementary Appendix 1). Clinicians had the option to complete the interview verbally or in writing. We recruited researchers and research coordinators with experience conducting research in the pediatric ED based on their participation in the Pediatric Emergency Care Applied Research Network and the Pediatric Emergency Medicine Collaborative Research Committee. Researchers and research coordinators were approached by email for a 4-item structured interview that could be completed via email or video conference call. Similar to clinicians, researchers, and research coordinators were asked to identify barriers to and facilitators of research enrollment in the pediatric ED (Supplementary Appendix 1). Additionally, researchers and research coordinators were asked about their institution’s policies around the use of a “warm hand-off,” in which a member of the clinical team introduced the research study to the potential participant before a research team member could approach the caregiver.^16^

### 2.3 Analysis

We analyzed quantitative data with descriptive statistics, including proportions for categoric data and medians and interquartile ranges for nonnormally distributed continuous data. Quantitative data were managed in RStudio (version 2023.12.1). We conducted a content analysis of qualitative data. With this approach, free-text data were deductively coded as facilitators and barriers, and important themes were synthesized. Saturation was determined based on recurrence and repetition. Qualitative data were managed in Atlas.ti (version 24.0). To maintain confidentiality, representative quotes are identified using numbers, with “C” if the participant was a clinician and “R” if the participant was a researcher or research coordinator. Quantitative and qualitative data from key stakeholders and experts were merged to develop a framework that highlights important concepts and findings from both quantitative and qualitative data sources.^13^ To do this, we grouped findings from both the quantitative and qualitative data sets and developed overarching thematic principles for each group.

## 3 Results

### 3.1 Caregivers

Overall, 368 caregivers were approached for participation, of whom 225 completed the survey (61.1% response rate). Table 1 shows the characteristics of caregiver participants, and Table 2 shows caregiver responses related to preferences for research recruitment and enrollment. The majority of caregiver respondents were female (72.4%), parents (85.3%), and aged between 25 and 44 years old (72.6%). Most caregivers perceived the times after clinician evaluation (79.8%) and after medical care was complete (82.1%) as the best for learning about research studies; some caregivers (16.5%-39.4%) perceived earlier times in the ED visit, including those before clinical care began, as good times to be approached about research opportunities. Caregiver expectations for incentive payment were proportionate to the degree of perceived risk of participation. Caregivers’ likelihood of participating in research was high but varied by type of research study, their perception of the impact of their research, and the context in which they were approached (Fig and Table 2).

**Table 1:** Characteristics of caregiver participants

Characteristic	No. (%) n = 225
Sex	
Female	163 (72.4)
Male	51 (22.7)
Age	
18-24 y	22 (9.8)
25-34 y	77 (34.2)
35-44 y	80 (35.6)
45-54 y	23 (10.2)
55 y or older	14 (6.2)
Missing	9 (4.0)
Race and ethnicity	
American Indian and Alaska Native	1 (0.4)
Asian	3 (1.3)
Hispanic	22 (9.8)
Multiracial	7 (3.1)
Non-Hispanic Black	42 (18.7)
Non-Hispanic White	132 (58.7)
Other race or ethnicity	2 (0.9)
Prefer not to say	8 (3.6)
Missing	8 (3.6)
Relationship to child	
Parent	192 (85.3)
Grandparent	10 (4.4)
Legal guardian	10 (4.4)
Other family	2 (0.9)
Missing	11 (4.9)
Child age	
<1 y	19 (8.4)
1-3 y	71 (31.6)
4-6 y	36 (16.0)
7-12 y	48 (21.3)
13-15 y	27 (12.0)
≥16 y	17 (7.6)
Missing	7 (3.1)

**Table 2:** Caregiver preferences for research recruitment and enrollment in the ED

Questionnaire item	No. (%)^a^
Which of these is a good time to be asked if you want to learn about a research study for you or your child?^b^	
When I check in to the ER at the front desk	36 (16.5)
When I am in the waiting room	83 (38.1)
When the nurse is checking my child in triage	45 (20.6)
When I am waiting to see the doctor for the first time	86 (39.4)
When I am waiting after the doctor has seen me	174 (79.8)
After the doctor has finished taking care of me	179 (82.1)
After I go home from the ER	139 (63.8)
By phone call^c^	30 (21.6)
By text^c^	74 (53.2)
By email^c^	90 (64.7)
By mailed letter^c^	35 (25.2)
Through the health record app^c^	42 (30.2)
If your doctor knows about a research study for you or your child, would it be ok for the researcher to discuss the study with you?	
Yes	198 (90.8)
No	19 (8.7)
I don’t want to be told about research while I’m in the ER^d^	8 (42)
I want my doctor to ask me first if I want to talk to the researcher^d^	10 (53)
I am likely or very likely to join a study where…^e^	
…I answer questions in an online survey	193 (88.5)
…I answer questions in an interview with a researcher	161 (73.9)
…researchers can get information from my child’s medical record	137 (62.9)
…my child would need to have their blood drawn	105 (48.2)
What do you think is a fair amount of compensation for being in each type of research study?^f^	
A survey that takes 30 min to fill out	$20 ($10, $30)
A 30-min interview with a researcher	$30 ($20, $40)
A study where my child has to get their blood drawn	$50 ($30, $100)
Is there anything else that you think we should know about what families think about research studies for children and families in the ER?^g^	
The need for information to be explained well	
*“Provide a lot of information” “The more information, the better”*	
The importance of acknowledging and respecting parent stress and worry during an ED visit	
*“People are tired and stressed.” “Use discretion when considering how and when to approach depending on how much is going on”*	
The potential benefit of learning and helping others through research participation.	
*“Impress that the child is not a guinea pig; this is cutting edge and could be beneficial. Maybe not in the immediate timeframe, but the future” “I would be willing to help in a research study that would help my family or someone else’s for free”*	

ED, emergency department.

a Overall 225 responses. The total for each question may vary because of the exclusion of nonresponses by the question.

b For each item, participants could select from a 4-point Likert scale response (Definitely not a good time, Not a good time, A good time, Definitely a good time to learn there is a research study). Responses shown are those who selected “A good time” or “Definitely a good time” to learn there is a research study.

c Item only asked if the participant indicated that after the ED visit was a good time to be approached about research (n = 139).

d Item only asked if a participant selected “No” (n = 19).

e For each item, participants could select from a 4-point Likert scale response (Very Unlikely, Unlikely, Likely, Very Likely).

f Responses shown are median (interquartile range).

g Prominent concepts and representative quotations from 26 free-text responses.

**Figure:**
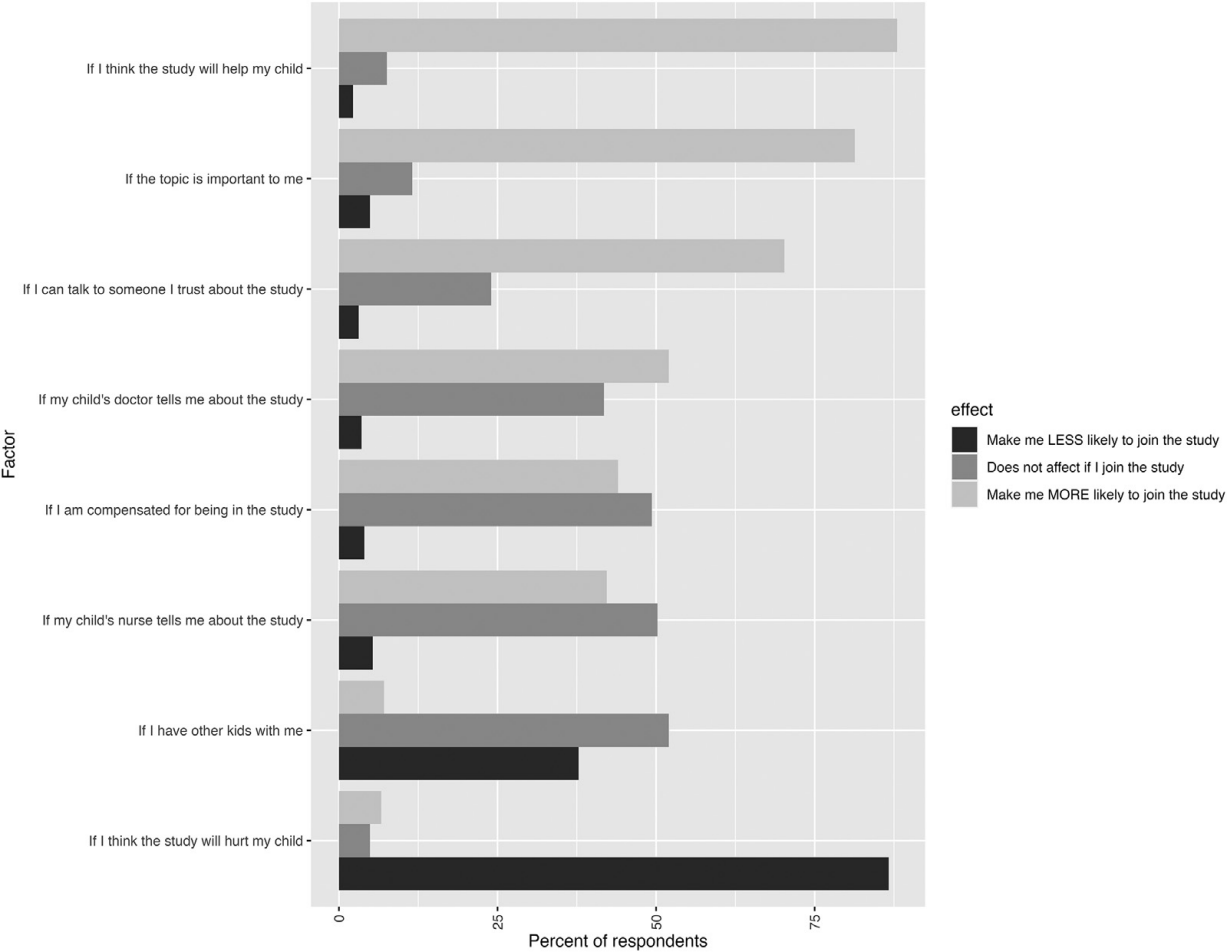
Caregiver perspectives on how factors may influence their decision to participate in research.

Twenty-six caregivers (11.6%) provided a response to the open-ended question assessing additional input regarding family perspectives on research with children and families in the pediatric ED. Three concepts were prominent in these free-text responses (Table 2): (1) the need for information to be explained well, (2) the importance of acknowledging and respecting caregiver stress and worry during an ED visit, and (3) the potential benefit of learning and helping others through research participation.

### 3.2 Clinicians, Researchers, and Research Coordinators

Eight ED clinicians (5 nurses, 2 physicians, and 1 support staff member; 100% response rate) and 11 researchers and research coordinators (8 physician-researchers and 3 research coordinators from 10 institutions; 69% response rate) completed structured interviews. Barriers and facilitators were identified at the level of the caregiver, clinician, and ED (Table 3). Researcher participants from 2 institutions described their institution’s requirement for a “warm hand-off,” although 1 of these participants noted an exception for minimal-risk research. Although not required at the other 9 institutions, some participants noted benefits from the “warm hand-off” process: “We have found that if there is a warm hand-off patients appear more enthusiastic about participation” (R10). However, others raised concerns, including that “the time spent asking a provider adds time to the length of stay, and also because providers do not always know about the study, they may not give the correct information….it also increases the provider workload, and having overburdened providers can impact the quality of care provided to all patients” (R11).

**Table 3:** Barriers and facilitators to pediatric ED research identified by clinicians, researchers, and research coordinators

Level	Type	Content	Illustrative Quotes^a^
Caregiver	Barriers	Stress related to the ED visit	Usually when families come in they are in a crisis of some sort…(R10)
		Ethical considerations	Doctors shouldn’t do it [enroll patients], [they are] too biased, the patient wants to please the doctor, [it] could lead to a power inequity and impact care (C6)
		Social context	Requiring a warm hand-off may cause us to miss patients who are discharged before the provider can introduce the study, which can lead to inequitable enrollment (especially true for patients speaking a language other than English because providers just would not introduce the study because it takes extra time to use the interpreter) (R11)
	Facilitators	Communication with a clinical team	It's extremely helpful when the provider introduces the research coordinator to the family before they pitch the study. The family trusts their care provider and that this study may be a good opportunity for them. (R2)
		Reading the room	RCs are highly trained in reading the room to understand if (approaching the family for research) is appropriate or not…RCs need to have emotional skills to read the room. (R3)
		Knowledge of the study	(It is important tobe) knowledgeable, compassionate research coordinators (R4)
		Research as an opportunity	There will be a spectrum of responses to research… some are incredibly grateful that we’re trying to improve care (R4)
Clinician	Barriers	Competing clinical priorities	As the ED becomes busier and the studies we are performing become more complicated, it’s harder to get buy-in from the clinical teams about the extra steps needed for research. When staff is stressed, they are less likely to want to take the time to fill out surveys or obtain clinical samples (especially new clinical blood draws). They can be short of research staff, which hurts the partnership. (R7)
	Facilitators	Partnership with clinical teams	It is essential to engage everyone (nurses and physicians) about why each study is taking place. There needs to be lots of build-up before the study rolls out, and champions within each group (MDs, RNs, RCs). There needs to be buy-in through education. And snacks for staff! (R4)
		Clinician knowledge of the study	We try to get into rooms early, so clinicians are available to answer any questions (R6)
System	Barriers	Clinical care is the priority	[The best time to approach for research is] after the attending comes in and all orders are put in so the patient is stable (laboratorie, results are pending) and just waiting; [the worst time is] on ED arrival, [when clinicians] don't have a history of what is going on and efficiency is slowed down (C2)
		ED environment	Time is a huge issue, as turnover is rapid and patients do not remain in the ED for research procedures very often. The other is the trust issue…it is hard to develop rapport and trust with a family in an acute setting. I think we try to get into rooms early, so we (clinicians) are available to answer any questions. (R6)
		RC availability	Enrolling is also more difficult when our research staff isn’t available. We’ve tried utilizing secure alerts as a way to know who might be eligible for a study and to remind staff to enroll when research staff is not available. Our more motivated research staff will often message providers via secure chat themselves when they get these alerts to try to help facilitate (though this has a negative impact on their work/life balance). (R7)
	Facilitators	Flexible recruitment processes	To overcome (limited overnight RC coverage), we implement a remote recruitment, or next-day recruitment process for all studies to ensure we give everyone the opportunity to participate in research. (R2)
		Culture of research	Having a culture in which research coordinators are viewed as part of the team and research is part of the care we provide (R3)

ED, emergency department; RC, research coordinator.

a Participant quotes are labeled with unique identifiers in which “C” represents clinicians and “R” represents researchers and research coordinators.

### 3.3 Framework

Table 4 presents a framework developed through the merging of qualitative and quantitative data collected from caregivers, clinicians, researchers, and research coordinators. The framework is composed of 5 key principles to support research recruitment and enrollment in the pediatric ED: (1) high-quality communication is essential; (2) discussions about research participation cannot be coercive; (3) partnership with clinical teams is necessary; 4) research teams must adapt to unpredictability; and (5) participation in research may be viewed as a potential opportunity by families. In addition to containing descriptive statements grounded in our data, the framework additionally integrates concepts that arose during data collection and analysis but that lack foundational data. Those concepts are highlighted as areas that should be considered in future research. These include the use of simulation activities to develop communication skills for research team members, the ethical considerations of the “warm hand-off” in facilitating introductions to research team members, and the role of conceptualizing research as an opportunity to enhance efforts for inclusivity in research recruitment.

**Table 4:** Framework for research with children and families in the ED

Principle 1. High-quality communication with families is essential Families who are receiving care in the ED often face high levels of stress and uncertainty. Not all families are interested in research, and family preferences regarding when and by whom they’d like to be approached about research participation vary. It is essential that research team members be able to assess the appropriateness of discussing research and to recognize and respond to families’ emotional needs. Research team members must also be knowledgeable about the research and be able to provide families with a clear rationale for the study, in addition to an explanation of the risks and benefits of participation. *Suggested area for future research: What is the role of simulation and role-playing exercises in developing communication skills among research team members?*
Principle 2. Discussions about research participation cannot be coercive As in any setting, research teams must not employ coercive strategies when engaging with families. Parent expectations for incentive or compensation payment are modest but proportionate to the degree of risk of participation. Some families may be more likely to participate when they are told about the opportunity for research by their treating physician. *Suggested area for future research: What are the relative risks and benefits of a warm hand-off between the clinical and research teams? How does this impact clinicians? How does this impact potential participants?*
Principle 3. Partnership with clinical teams is necessary Research may be perceived as an added burden on clinical teams. In addition to efforts to minimize the impact on clinical care, research teams can partner with clinical teams by providing clinical teams with education on the rationale and importance of the study, including how it is expected to benefit clinical care, and by sharing study outcomes when the research is complete.
Principle 4. Research teams must be able to adapt to unpredictability Clinical care of the child is the first priority in the ED, and research teams must be flexible to work around the unpredictable flow of ED care. Research teams can minimize their impact on clinical care by checking in with the clinical team before approaching the family and by waiting to approach the family until the end of the ED visit when possible.
Principle 5. Participation in research may be viewed as a potential opportunity for families Although not all families will want to participate in research, many appreciate the opportunity to contribute and view research participation as a way to help others. *Suggested area for future research: If research is conceptualized as an opportunity or as part of the care provided in the ED, how does this impact research participants? How does this impact inclusivity in recruitment and enrollment?*

ED, emergency department.

## 4 Limitations

This research is subject to limitations. We approached a convenience sample of caregivers and clinicians based on research team availability, although we designed our recruitment schedule to ensure recruitment occurred at most times of day and night over all days of the week. Our caregiver response rate was appropriate for the study design, but we are unable to characterize nonrespondents or determine how their nonparticipation biased our data. We chose to conduct questionnaires, rather than individual interviews, with caregivers in order to enhance our ability to capture the perspectives of a large number of caregivers within the resource limitations of the study. Future qualitative work to explore the in-depth perspectives of caregivers is needed. Our qualitative responses included text from email responses and field notes from verbal responses; this may have led to subtle differences in interpretations between the 2 types of data. We also did not conduct member-checking of our proposed framework. Additionally, we only recruited clinicians and caregivers from our single center. Although this allowed us to capture the perspectives of key stakeholders in an environment without substantial prior pediatric ED research, these perspectives may not be generalizable. Further, although our ED has limited experience with conducting research with children and families, our institution is an academic medical center, and, thus, caregivers and clinicians may still have experience with research, and their perspectives may not be the same as those encountered in community settings. As emergency research with children expands to community EDs, it will be essential to gather and incorporate perspectives from patients and clinicians in those settings. Finally, although the racial and ethnic demographic characteristics of the caregivers we recruited were similar to those of the overall patient population at our institution, we only recruited caregivers who used English for medical care. As such, our findings do not represent the important perspectives of caregivers who use languages other than English for medical care.

## 5 Discussion

In this mixed-methods research, we engaged key stakeholders and experts to develop a pragmatic framework for the recruitment and enrollment of children and families in ED research. Our framework contains concepts foundational to research enrollment in all settings while highlighting unique considerations for children and families in the ED and noting areas in need of ongoing research. This framework has the potential to support protocol development and implementation for researchers, especially if they themselves, or the institutional stakeholders they are engaging, do not have prior experience recruiting children and families for research in the ED.

Our results demonstrate that there is no “one size fits-all” approach that works best for approaching caregivers about research in the pediatric ED. Instead, research teams must be flexible to adapt to the unpredictable nature of the ED while simultaneously assessing the caregiver’s interest in and emotional availability for research recruitment at various times during ED visits. Although most caregivers preferred to be approached after clinical care was initiated or completed, this was not universally true. Some caregivers were willing to be approached earlier in the ED visit, but assessing this willingness requires research team members to employ high-quality, empathic communication. Additionally, our results support existing literature demonstrating that caregivers of acutely ill children, both in ED and intensive care settings, often view participation in research as an opportunity to help others.^15^^,^^17^^,^^18^ This is further supported by the fact that, though participants of all types acknowledged that caregivers experience stress related to ED visits, this was not noted by caregivers to necessarily preclude research enrollment. It is then reasonable to conclude that with proper communication, research can be conducted with children and families in the ED setting. There is growing interest in using simulation to teach communication skills for research teams engaging with traditionally under-considered populations of potential research participants, including older adults and individuals who use languages other than English.^19^^,^^20^ Such simulation and role-playing exercises may be effective in developing communication skills to support research team members in effectively and empathically approaching children and families about research in the ED setting.

There is continued uncertainty regarding best practices for partnering with clinical teams when recruiting children and families in research in the ED. Although rarely required by institutions, clinicians and researchers often believed that an introduction from the clinical team could facilitate a family’s interest in research due to the typically trustful relationship between clinicians and patients. However, some also noted concerns with this approach, including the risk of coercion, the added workload required of clinicians, and the potential for clinician bias in participant selection and research “gate-keeping.”^16^^,^^21^ Additionally, some institutions have IRB policies that prohibit “cold-calling,” or the practice of interacting with potential research participants with no previously existing clinical relationship.^16^ Policies against cold-calling have been developed to protect patient privacy but may conflict with principles of research ethics.^16^ However, some research, including our own, indicates caregivers of pediatric ED patients are willing to be approached about research by nonclinical research team members.^17^ Though there are differences between institutions, expert researchers and research coordinators agree that emphasis should be placed on research enrollment processes that minimize disruptions to clinical care. There remains a clear need for further research to determine how policies that require clinicians to introduce research opportunities are perceived by patients and potential participants, and their impact on participant autonomy, clinical care, and opportunities for equitable and inclusive research participation.^16^

Our results support existing literature demonstrating that caregivers of acutely ill children, both in ED and intensive care settings, often view participation in research as an opportunity to help others.^15^^,^^17^^,^^18^ This is important: IRBs, who have the critical role of protecting research participants, rightly place emphasis on minimizing and clearly communicating any potential risk to participants. However, adequate attention should be placed on potential benefits, as some caregivers find that research participation serves an altruistic benefit. This is particularly true for minimal-risk research, where participants face little to no possibility of harm. Yet in such settings, the risk may be inaccurately perceived as high if the informed consent process required by the institution is lengthy and overwhelming, potentially undermining the potential for altruistic benefit.^8^ Instead, there should be a balance between respecting caregivers’ interest in altruism while also adequately informing patients. Enhanced, interactive, and plain language informed consent processes have the potential to engage potential participants in meaningfully understanding the actual risks, benefits, and requirements of research participation.^22^, ^23^, ^24^ Efforts such as these serve as a foundation for developing methods of recruitment and obtaining informed consent that center the potential participant’s ability to reflect on whether and how the proposed research aligns with their values.

In conclusion, based on the synthesis of data collected through multiple methods from experts and key stakeholders, our framework provides key considerations and areas in need of future study for conducting research in the pediatric ED. It additionally serves as a foundation for researchers embarking on their own scientific inquiry to improve health outcomes for acutely ill and injured children.

## Author Contributions

CKG and RF conceptualized and designed the study. AR, CKG, AM, and RF designed the data collection instrument. AR, DMP, and JM participated in data collection. CKG supervised data collection. AR and CKG carried out data analyses. MT and RF contributed to data analysis. AR drafted the initial manuscript. All authors critically reviewed and revised the manuscript for important intellectual content, approved the final manuscript as submitted, and agreed to be accountable for all aspects of the work.

## Funding and Support

Colleen K. Gutman was supported by 10.13039/100000002NIH/10.13039/100006108NCATS KL2TR001429 and NIH/NIMHD K23MD018639. The NIH, NCATS, and NIMHD had no role in the design and conduct of the study.

## Conflict of Interest

All authors have affirmed they have no conflicts of interest to declare.
