# Supplementary material for: Development of a Framework for the Enrollment of Children and Families in Emergency Department Research
Source: J Am Coll Emerg Physicians Open. 2025 Jan 9;6(1):100018. doi: 10.1016/j.acepjo.2024.100018 (PMC11852677; doi:10.1016/j.acepjo.2024.100018)
Supplement: Supplementary Appendix 1 [file mmc1.docx]

**Appendix 1: Study Instruments**

**1. Caregiver questionnaire**

Scientists conduct research studies to find the best way to help kids who are sick. Participating in research has several benefits like helping others, learning new things, and helping all kids be healthy. It's up to you or your child if you want to be part of a research study. Right now, we're trying to figure out what things make it easier or harder for people to take part in research in the emergency department for kids.

Before your child joins a research study, a researcher meets with you to tell you about the research and to get your permission to join.

The next questions are about when and how you would want to be told that you could learn about a research study. This is not about when you would want to talk to the researcher about the details about the study, just when and how you would want to be told that you could talk to the researcher.

Are each of these good times to be asked if you want to learn about a research study for you or your child?

When I check in to the ER at the front desk

Yes, this is a good time to be asked if I want to learn about a research study

No, this is not a good time to be asked if I want to learn about a research study

When I am in the waiting room

Yes, this is a good time to be asked if I want to learn about a research study

No, this is not a good time to be asked if I want to learn about a research study

When the nurse is checking my child in triage

Yes, this is a good time to be asked if I want to learn about a research study

No, this is not a good time to be asked if I want to learn about a research study

When I am waiting to see the doctor for the first time

Yes, this is a good time to be asked if I want to learn about a research study

No, this is not a good time to be asked if I want to learn about a research study

When I am waiting after the doctor has seen me

Yes, this is a good time to be asked if I want to learn about a research study

No, this is not a good time to be asked if I want to learn about a research study

After the doctor has finished taking care of me

Yes, this is a good time to be asked if I want to learn about a research study

No, this is not a good time to be asked if I want to learn about a research study

After I go home from the ER

Yes, this is a good time to be asked if I want to learn about a research study

How would you want to be told about a research study after you leave the ER?

A phone call

A text

An email

A mailed letter

Through the health record app

Another way: ____________

No, this is not a good time to be asked if I want to learn about a research study

If your doctor knows about a research study for you or your child, would it be ok for the researcher to discuss the study with you?

Yes

No

What is your reason for saying no?

I don’t want to be told about research while I’m in the ER

I want my doctor to ask me first if I want to talk to the researcher

Another reason: __________

How would each of these things affect your decision to be in a research study in the ER?

If the research is on a topic that is important to me or my child

Makes me LESS likely to join the study

Does not affect if I join the study

Makes me MORE likely to join the study

If I am paid or compensated for being in the research study

Makes me LESS likely to join the study

Does not affect if I join the study

Makes me MORE likely to join the study

If I think the research study could help me or my child

Makes me LESS likely to join the study

Does not affect if I join the study

Makes me MORE likely to join the study

If I think the research study could hurt me or my child

Makes me LESS likely to join the study

Does not affect if I join the study

Makes me MORE likely to join the study

If I have time to talk to someone I trust before making the decision

Makes me LESS likely to join the study

Does not affect if I join the study

Makes me MORE likely to join the study

If I have other children with me

Makes me LESS likely to join the study

Does not affect if I join the study

Makes me MORE likely to join the study

If my doctor tells me about the research study

Makes me LESS likely to join the study

Does not affect if I join the study

Makes me MORE likely to join the study

If my nurse tells me about the research study

Makes me LESS likely to join the study

Does not affect if I join the study

Makes me MORE likely to join the study

How likely are you to join a research study where….

…you answer questions in an online survey

Very unlikely

Unlikely

Likely

Very likely

…you answer questions in an interview with a researcher

Very unlikely

Unlikely

Likely

Very likely

…researchers can get information from your child’s medical record

Very unlikely

Unlikely

Likely

Very likely

…your child would have their blood drawn

Very unlikely

Unlikely

Likely

Very likely

Sometimes researchers give families pay or compensation for being in a research study. What do you think is a fair amount for each type of research study? (Example: $10, $20, $30, $40, etc)

Fair amount of compensation for filling out a survey that takes 30 minutes: ___

Fair amount of compensation for doing a 30 minute interview with a researcher: ___

Fair amount of compensation if my child has their blood drawn for a research study: ___

Is there anything else that you think we should know about what families think about research studies for children and families in the ER?

Demographic Questions

What is your relationship to the child that is here in the ER today?

Parent

Grandparent

Other family member

Legal guardian

Other

How old are you?

18-24 years old

25-34 years old

35-44 years old

45-54 years old

More than 54 years old

How old is the child that is with you and being taken care of in the ER today?

Less than 1 year old

1-3 years old

4-6 years old

7-12 years old

13-15 years old

16 years or older

What is your race and ethnicity? Select all that apply.

American Indian or Alaska Native

Asian

Black or African American

Hispanic

Native Hawaiian or Other Pacific Islander

White

Other: _________

Prefer not to say

What is your gender?

Female

Male

Non-binary

Prefer to self describe: _________

Prefer not to say

**2. Clinician structured interview**

When do you think is the best time for parents and caregivers to be approached by someone about research? When are the worst times? Why?

Who do you think is the best person to approach a parent or caregiver about research? Who should not do it? Why?

Have you had any experiences telling patients about research opportunities? What went well? What didn’t? Why?

Is there anything else you think is important for researchers to think about when they are coming up with plans to talk to families about research during the pediatric ED visit?

**3. Researcher and research coordinator structured interview**

What is the process at your institution for approaching patients and caregivers about research in the pediatric ED?

What do you find works well when approaching patients and caregivers about research in the pediatric ED? What doesn’t work well? Why?

What barriers have you faced to conducting research with patients and caregivers in the pediatric ED? What strategies have you used to address those barriers?

Some institutions require a “warm hand-off", where someone from the clinical team has to ask the patient/caregiver for permission for a someone from research to talk to them (before someone from research can approach the patient/caregiver). Is this something that is required at your institution? If so, how do you navigate this? If not, was this ever a policy at your institution? What led to the change?
